# FOXO1 promotes tumor progression by increased M2 macrophage infiltration in esophageal squamous cell carcinoma

**DOI:** 10.7150/thno.45261

**Published:** 2020-09-16

**Authors:** Ying Wang, Zhaojie Lyu, Yanru Qin, Xia Wang, Liangzhan Sun, Yu Zhang, Lanqi Gong, Shayi Wu, Shuo Han, Ying Tang, Yongxu Jia, Dora Lai-Wan Kwong, NgarWoon Kam, Xin-Yuan Guan

**Affiliations:** 1 Department of Clinical Oncology, The University of Hong Kong-Shenzhen Hospital, Shenzhen, China.; 2 Department of Clinical Oncology, Li Ka Shing Faculty of Medicine, The University of Hong Kong, Hong Kong.; 3 Department of Radiation Oncology, Sun Yat-Sen University Cancer Center, Guangzhou, China.; 4 State Key Laboratory of Oncology in South China, Sun Yat-Sen University Cancer Center, Guangzhou, China.; 5 Department of Clinical Oncology, the First Affiliated Hospital, Zhengzhou University, Zhengzhou, China.; 6 School of Biomedical Sciences, Li Ka Shing Faculty of Medicine, The University of Hong Kong, Hong Kong.; 7 Shenzhen Institutes of Advanced Technology, Chinese Acadamy of Science, Shenzhen, China.

**Keywords:** FOXO1, M2 macrophage, esophageal squamous cell carcinoma, cancer progression

## Abstract

**Objective:** The transcription factor forkhead box protein O1 (FOXO1) is critical for regulating cytokine and chemokine secretion. However, its function in the tumor microenvironment (TME) remains largely unexplored. In this study, we characterized the prognostic value of FOXO1 and the interaction between tumor-derived FOXO1 and M2 macrophages in esophageal squamous cell carcinoma (ESCC).

**Methods:** FOXO1 expression and macrophage infiltration in clinical samples and mouse models were quantified using quantitative real-time polymerase chain reaction (qRT-PCR) and immunohistochemistry staining. Western blotting, qRT-PCR, and enzyme-linked immunosorbent assay were used to evaluate chemokine ligand 20 (CCL20) and colony stimulating factor 1 (CSF-1) expression in FOXO1(+) and FOXO1(-) tumor cells. Macrophage phenotypes were determined using qRT-PCR, flow cytometry, and RNA sequencing. Transcriptional activity was measured using chromatin immunoprecipitation (ChIP)-qPCR. Tumor viability was investigated using XTT proliferation and foci formation assays.

**Results:** FOXO1 upregulation in tumor tissues was found to drive the polarization of M0 macrophages and infiltration of M2 macrophages into the TME, resulting in worse prognosis in ESCC patients. CSF-1, a vital factor inducing M0-to-M2 polarization, was upregulated via a FOXO1-mediated mechanism. RNA sequencing results corroborated that the FOXO1-induced macrophages exhibited similar molecular signatures to the IL4-stimulated M2 macrophages. The transwell assays showed that FOXO1 promoted the migration of M2 macrophages via CCL20 secretion, which could be inhibited using an anti-CCL20 antibody. FOXO1(+) tumor-induced M2 macrophages promoted tumor proliferation via the FAK-PI3K-AKT pathway and the PI3K inhibitor could effectively impede the oncogenical process.

**Conclusions:** FOXO1 facilitated M0-to-M2 polarization and the recruitment of M2 macrophages in the TME via the transcriptional modulation of CCL20 and CSF-1. Our data deciphered the FOXO1-dependent mechanism in M2 macrophage infiltration in the TME of ESCC, which has implications for the development of novel prognostic and therapeutic targets to optimize the current treatment against ESCC.

## Introduction

Esophageal cancer ranks as the sixth leading cause of cancer-related mortality worldwide 1 and esophageal squamous cell carcinoma (ESCC) has a high global incidence, especially in central and southern Asia, accounting for approximately 80% of the total cases worldwide 2. Despite recent advances in therapeutics against ESCC, the 5-year survival rate still ranges from 9% to 27.1% 3-5 and emerging therapeutic targets are in high demand. Esophageal mucosa with chronic irritation and inflammation has shown a tendency to develop into ESCC 2; therefore, it is important to identify and characterize the crucial role of the tumor microenvironment (TME) during disease progression. However, the TME is a highly heterogeneous ecosystem that consists of multiple cellular components, including infiltrating lymphocytes, fibroblasts and endothelial cells. Among these different cell types, infiltrating tumor-associated macrophages (TAMs) have attracted great attention because they are a poor prognostic factor in many cancer types 6. There are two well-recognized subtypes of macrophages, which are classically activated macrophages (M1) and alternatively activated macrophages (M2). TAMs have been reported to share similar functional and phenotypic characteristics to those of M2 macrophages. For example, both TAMs and M2 macrophages can exert immunosuppressive effects and promote angiogenesis after activation by IL10 or Arg1 7. Therefore, targeting infiltrating TAMs could serve as a promising target in cancer therapy.

Forkhead box protein O1 (FOXO1) belongs to the forkhead box O (FOXO) family of transcription factors. Recent studies have revealed that FOXO1 can regulate several downstream pro-inflammatory factors. For example, FOXO1 was shown to significantly enhance the production of chemokine ligand 20 (CCL20) to promote lymphocyte chemotaxis 8 and to manipulate IL10 secretion during chronic inflammation 9.

The role of FOXO1 in inflammatory and metabolic disorders has been widely investigated; however, its function in cancer progression remains unclear. FOXO1 deficiency is correlated with worse clinical outcomes in breast cancer 10, 11, bladder cancer 12 and cervical cancer 13 because it can induce cell cycle arrest and apoptosis 14. However, in other malignancies, such as urothelial carcinoma 15, FOXO1 amplification facilitates tumor growth and metastasis, leading to poor prognosis. Because the role of FOXO1 in different malignancies remains controversial, a more comprehensive understanding of the FOXO1-related mechanism would help reevaluate its potential value in ESCC diagnosis and treatment.

## Methods

### Cell lines and incubation

Human ESCC cell lines KYSE180 and KYSE510 were cultured in RPMI1640 with 10% fetal bovine serum (FBS) and 1% penicillin-streptomycin. Human monocyte cell line THP-1 cells (kindly provided by Prof Professor CHUI Yiu Loon in Department of Chemical Pathology, The Chinese University of Hong Kong) were cultured in RPMI1640 with 10% heat-inactivated FBS and 1% penicillin-streptomycin. All the cell lines were incubated at 37 °C and 5% CO_2_ in a humidified atmosphere.

### Establishment of stable FOXO1 overexpression and FOXO1 knockdown cell lines

The human FOXO1 plasmid and its negative control (vector) plasmid were purchased from Genecopoeia. The FOXO1-expressing and vector-expressing lentiviruses were produced from 293FT cells transfected with either FOXO1 or vector plasmid using ViraPower Lentiviral Packaging Mix (Invitrogen) and Lipo2000 (Invitrogen). The lentiviruses were later co-cultured with KYSE180 and KYSE510 to establish stable FOXO1(+) and FOXO1(-) ESCC cell lines.

Based on *the FOXO1* sequence (NM_002015.4), two shRNAs were designed and the sequences were as follows:

sh*FOXO1*-1 (CCGGATTCTGCACACGAATGAACTTTCAAGAGAAGTTCATTCGTGTGCAGAATTTTTTG); sh*FOXO1*-2 (CCGGACTTATTGTCCTGAAGTGTCTTCGAAGACACTTCAGGACAATAAGTTTTTTG).

The *FOXO-1* shRNA and scrambled shRNA were constructed using pLKO.1 puro purchased from Addgene (Plasmid #8453). KYSE180-FOXO1(+) and KYSE510-FOXO1(+) tumor cells were transfected with *FOXO1*-specific shRNA or scrambled shRNA plasmid to establish FOXO1 knockdown cell lines (FOXO1(+)-sh1, FOXO1(+)-sh2, and FOXO1(+)-ctl).

### The model of macrophages polarization

THP-1 cells were differentiated into an intermediate stage M0 under the stimulus of phorbol 12-myristate 13-acetate (PMA). Then, the M0 macrophages were polarized into M2 macrophages via IL4 and IL13 stimulation.

### RNA extraction and quantitative real-time polymerase chain reaction (qRT-PCR)

Cells were resuspended and lysed in Trizol (Invitrogen) and complementary DNA (cDNA) was synthesized using a reverse transcription-PCR Kit (Roche) according to the manufacturer's instructions. qRT-PCR was performed using SYBR Green PCR Kit (Applied Biosystems) on a 384-well plate with an ABI PRISM 7900 Sequence Detector (Applied Biosystems). The results were analyzed using the ABI SDS v2.4 software (Applied Biosystems). The sequences of primers used are listed in Table S1.

### Western blotting and antibodies

Equal amounts of protein were loaded and separated using sodium dodecyl sulfate-polyacrylamide gel electrophoresis (SDS-PAGE). After transfer to a polyvinylidene difluoride membrane, the proteins were blocked with 5% bovine serum albumin (BSA) and incubated in primary antibodies at 4 °C overnight. Subsequently, horseradish peroxidase (HRP)-conjugated secondary antibody was used to incubate the samples for 2 h. The targeted proteins were detected and visualized with an enhanced chemiluminescence system (GE Healthcare) and X-ray film (GE Healthcare). Beta-actin was used as a loading control. The antibodies used are listed in Table S2.

### Enzyme-linked immunosorbent assay (ELISA)

ESCC cells were seeded in 6-well plates at a density of 1 × 10^6^ cells per well and incubated for 48 h. The supernatant was collected to detect the secretion of CCL20 and CSF-1. CCL20 ELISA kit (DM3A00) and CSF-1 ELISA kit (DMC00B) were purchased from R&D systems. ELISA was performed as per manufacturer's instructions.

### Flow cytometry

Cells were collected, washed, and incubated for 30 min at 4 °C with florescence-conjugated antibodies. To facilitate intracellular staining, cells were fixed and permeabilized with a fixation/permeabilization solution kit (BD Cytofix/Cytoperm) and 1% paraformaldehyde (PFA). The results were analyzed using the FlowJo 10.7 software program. The antibodies are listed in Table S2.

### Chromatin immunoprecipitation (ChIP)qPCR

A total of 8 × 10^6^ ESCC cells were seeded in a Petri dish and harvested in PBS after formaldehyde cross-linking. After centrifugation, protease inhibitor-containing SDS lysis buffer was added to the cell pellet. The mixture was sonicated to shear chromatin to an average length ranging from 200 to 500 base pairs. The ChIP assay was performed according to the manufacturer's instructions (MilliporeSigma, EZ-ChIP, Cat: 17-371). The input group accounted for 1% of the total DNA, while the IgG and FOXO1 groups were added with their ChIP grade antibodies using the suggested concentration from the manufacturers. After purification, qPCR was performed to detect the protein binding sites of the DNA samples. The calculation of the ChIP signal is % input = 1% × 2 ^ (CT_input_ - CT_sample_). The sequences of the primers were as follows: forward: CSF-1 promoter forward: CCCTTGGGACGATCATAGA and CSF-1 promoter reverse: GTCTTCCTAGTCACCCTCTGT.

### Tissue microarray (TMA)

A TMA containing 144 pairs of ESCCs (tumor and non-tumor tissues) from Linzhou Cancer Hospital (Henan, China) was constructed according to a previously described method 16. The tissue samples used in the present study were approved by the Committee for the Ethical Review of Research Involving Human Subjects at Zhengzhou University. The expression of FOXO1 was assessed by three independent investigators. There was no obvious difference between the percentage of stained cells and staining intensity; therefore, the immunoreactivity of FOXO1 was determined based on negative and positive staining.

### Immunohistochemistry (IHC) and immunofluorescence (IF)

Tissues (5 µm) were de-paraffinized in xylene and rehydrated with ethanol before antigen retrieval. For IHC, endogenous peroxidase (Dako) and protein blocking solution (Dako) were used before incubation of primary antibodies. Subsequently, EnVision Plus System-HRP (DAB; DAKO) was used according to the manufacturer's instructions and counterstaining was performed using Mayer's hematoxylin. For immunofluorescence (IF), 5% BSA was used as the blocking buffer before incubation with primary antibodies. DAPI (Invitrogen) was used to counterstain the tissues before mounting with fluorescent mounting medium (Dako). The antibodies used are listed in Table S2.

### Macrophage migration assay

After *in vitro* polarization of THP-1 cells, the migration assay was performed using 6.5 mm transwell plates with 5.0 µm pore inserts. FOXO1(+) or FOXO1(-) tumor cells were placed on the bottom of the lower chamber in a 24-well plate as a chemoattractant and M0 or M2 macrophages were added to the upper transwell inserts (Corning, Cat: 09717050) and incubated for 48 h at 37 °C and 5% CO_2_. To inhibit the effect of CCL20 secretion, tumor cells were incubated with α-CCL20 antibody (R&D Systems, Minneapolis, MAB360, USA) prior to the migration assay. For the M2 macrophage migration assay induced with the CCL20 recombinant (Peprotech, 300-29A), M2 macrophages were plated in the upper inserts and CCL20 recombinant was added to the bottom wells. After 48 h, the transwell inserts were removed from the plate and washed three times with PBS. Then, the remaining cells on the top of the membrane were wiped off with a cotton-tipped applicator. A sample of 4% PFA was used to fix the transwell inserts for 15 min. The inserts were immersed in 1% crystal violet for at least 15 min for staining and then dipped into distilled water to remove excess. The migration results were quantified using ImageJ.

### Transwell co-culture assay of M0 macrophages and tumor cells

Indirect co-culture assay was performed using 3.0 µm cell culture inserts (Corning, Cat: 353492). M0-polarized THP-1 cells were seeded in the upper insert and FOXO1(+) or FOXO1(-) tumor cells were seeded into the bottom wells in the presence of PMA. Macrophages were then collected and stained with M2 macrophage markers (CD68 and CD163) to identify the phenotypic changes busing flow cytometry. To inhibit the effect of CSF-1, tumor cells were incubated with the α-CSF-1 antibody (LifeSpan BioSciences; LS-C104656) prior to the co-culture assay.

### *In vitro* tumorigenic assays in the presence of conditioned medium from M2 macrophages

For the foci formation assay, parental ESCC cells were seeded in 6-well plates and cultured with M2 conditioned medium or complete medium (CM). After 7-day culture, the total number of colonies was counted after fixation and staining. For the XTT assay, 1 × 10^3^ cells in serum-free medium with M2 conditioned medium or CM were seeded in 96-well plates. The cell growth rate was determined using the XTT kit (Roche Applied Science) according to the manufacturer's instructions. The optical density value for each well was read at 450 nm using an automated microplate reader (Sunrise, Tecan, Switzerland).

### Wound healing experiment

Parental ESCC cells were plated in 6-well plates. After 24 h, a scraped cell-free area was made using a micropipette tip (200 µL) and M2 conditioned medium or the same percentage of CM was added. Wound closure was observed after 24 and 48 h and analyzed using the ImageJ software program.

### Tumor migration assay

The tumor migration assay was conducted using 8 µm culture inserts (Corning, Cat: 353097). Parental tumor cells were seeded into the upper inserts in the presence of conditioned medium from M2 macrophages, while 10% FBS RPMI 1640 medium was placed in the bottom chambers. After 24 or 48 h of incubation, the migrated cells across the membrane were stained and counted.

### Establishment of a xenograft-transplanted mouse model of ESCC

A total number of 3 × 10^6^ FOXO1(+)/FOXO1(-) tumor cells were injected into both flanks of nude mice subcutaneously. After four weeks, mice were sacrificed under anesthesia. One part of the tumor tissue was collected and embedded in paraffin for immunostaining to qualify macrophage infiltration, and the other part was lysed in Trizol for RNA extraction.

A total number of 3 × 10^6^ parental tumor cells were injected into both flanks of nude mice subcutaneously. After two weeks, 20 μL concentrated M2 conditioned medium and serum-free medium (control group) were injected into the tumor mass subcutaneously separately every three days. Tumor volumes were carefully monitored every week for 3-5 weeks to assess the tumor-promoting effect of M2-derived cytokines. Tumor size was measured according to the formula: Volume = Length × Width^2^/2.

### Statistical analysis

For clinical data, demographic characteristics were shown using descriptive statistics. The correlation between FOXO1 expression and clinicpathological features was assessed using Pearson's chi-square test. Survival curves were derived using the Kaplan-Meier method and assessed univariately using the log-rank test, with a significance level set at two-sided 0.05. The above steps were performed using the statistical software SPSS version 20.0. The analysis of 22 types of immune cell infiltration was conducted using the CIBERSORT algorithm 17.

## Results

### Overexpression of FOXO1 indicates poor prognosis in ESCC patients

**Figure 1:**
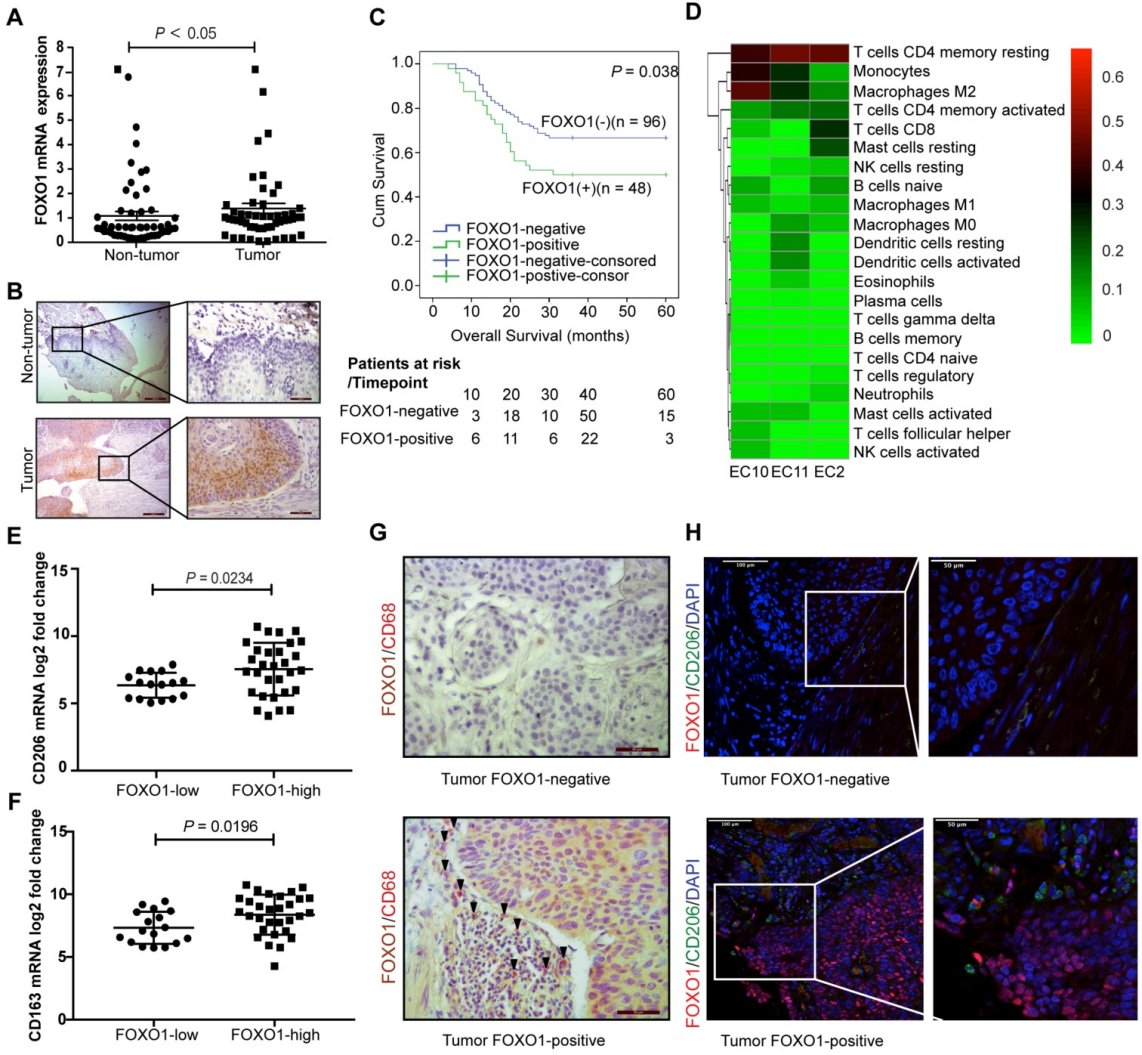
Overexpression of FOXO1 correlated with poor survival outcomes and high M2 macrophages infiltration in ESCC. (A) The relative expression of FOXO1 in 52 paired ESCC tumor and non-tumor samples was detected using qRT-PCR (*P* < 0.05). (B) Representative images for IHC staining showed FOXO1 expression in the ESCC tumor and non-tumor tissues. Scale bar, 200 µm (left) and 50 µm (right). (C) Kaplan-Meier curves for Overall survival (OS) showed that FOXO1-positive patients (n = 48) had worse prognosis than FOXO1-negative patients (n = 96) (*P* = 0.038). (D) The heat map of 22 types of immune cells infiltrating three ESCC tumor tissues analyzed using RNA-seq. The columns represent each patient sample and the proportions of the immune cells are shown as the color intensity. Red represents high density and green indicates low density. (E-F) ESCC patients in TCGA program (n = 47) were divided into two groups based on their FOXO1 expression. The mRNA expression of CD206 was overexpressed in the FOXO1-high group (n = 30) compared to the FOXO1-low group (n = 17) (*P* = 0.0234) (E). The mRNA expression of CD163 was overexpressed in the FOXO1-high group compared to the FOXO1-low group (*P* = 0.0196) (F). (G) Representative images of double IHC staining with FOXO1 (brown) and CD68 (red) in the FOXO1-positive and FOXO1-negative tumor tissues. Scale bar, 50 µm. (H) Representative images of IF staining with FOXO1 (red) and CD206 (green) in the FOXO1-positive and FOXO1-negative tumor tissues. Scale bar, 100 µm (left) and 50 µm (right).

RNA sequencing was performed on three paired ESCC tumor and non-tumor tissues to identify tumor-specific genetic profiles. The Venn diagram showed that FOXO1 was upregulated in all three ESCC tumor tissues (Figure S1A). To further corroborate the overexpression of FOXO1 in ESCC, 52 paired ESCC samples were analyzed with qRT-PCR. The results revealed that FOXO1 was frequently upregulated in tumor tissues compared to the non-tumor counterparts (*P* < 0.05, Figure 1A). IHC results also showed that FOXO1 was consistently overexpressed at the tumor margin (Figure 1B and Figure S1B). To validate the correlation between FOXO1 expression and prognosis in ESCC patients, a TMA consisting of 144 ESCC patient tumor tissues was analyzed. Based on FOXO1 expression, patients were divided into FOXO1-positive (n = 48, 33.3%) and FOXO1-negative (n = 96, 66.7%) groups. Patients with incomplete clinical and pathological data were excluded from the analysis. Kaplan-Meier survival analysis showed that FOXO1-positive patients had poorer clinical outcomes than FOXO1-negative patients (*P* = 0.038, Figure 1C). The clinicopathological analysis indicated that distant metastasis was also associated with FOXO1 expression (Pearson's chi-square test, *P* = 0.013). In contrast, age, pathologic T and N stages, pathology type, invasion level, differentiation, and gender exhibited no significant correlation with FOXO1 expression (Table 1). Multivariate Cox regression analysis suggested that FOXO1 was an independent prognostic factor with age and pathological N stage in ESCC patients (Table 2).

### M2 macrophage infiltration in ESCC tumor tissues

A total of 22 types of immune cell infiltration were quantified from the RNA sequencing data of three ESCC tumors using CIBERSORT. We found that CD4 memory resting T cells, M2 macrophages, and monocytes were the top three infiltrating immune cells in ESCC (Figure 1D). To further validate the infiltration of these immune cells, RNA sequencing data of ESCC from the Cancer Genome Atlas (TCGA) database was analyzed using CIBERSORT and yielded a similar result, with M2 and M0 macrophages and CD4 memory resting T cells being the top three largest immune cell subpopulations in the tumor tissues (Figure S1C). Moreover, we detected the expression of specific markers for these highly enriched immune cells in the FOXO1-high and FOXO1-low groups. There was no significant difference in the expression of pan-macrophage marker CD68 (*P* = 0.249), CD4 memory resting T cell marker CCR7 (*P* = 0.305), and monocyte marker CD14 *(P* = 0.650, Figure S1D), whereas M2 macrophage marker CD206 was significantly upregulated in the FOXO1-high group* (P* = 0.0254, Figure 1E). The infiltration of M2 macrophages was further confirmed by the drastically increased expression of CD163, another M2 macrophage-specific marker, in the FOXO1-high group (*P* = 0.0196, Figure 1F). Pathological assessment of the tumor tissues revealed that more CD68+ macrophages, especially CD206+ M2 macrophages, infiltrated the tumor stroma when tumor cells that highly expressed FOXO1 were in the marginal area (Figure 1G-H).

### FOXO1 promotes M2 macrophage infiltration in xenograft tumors

**Figure 2:**
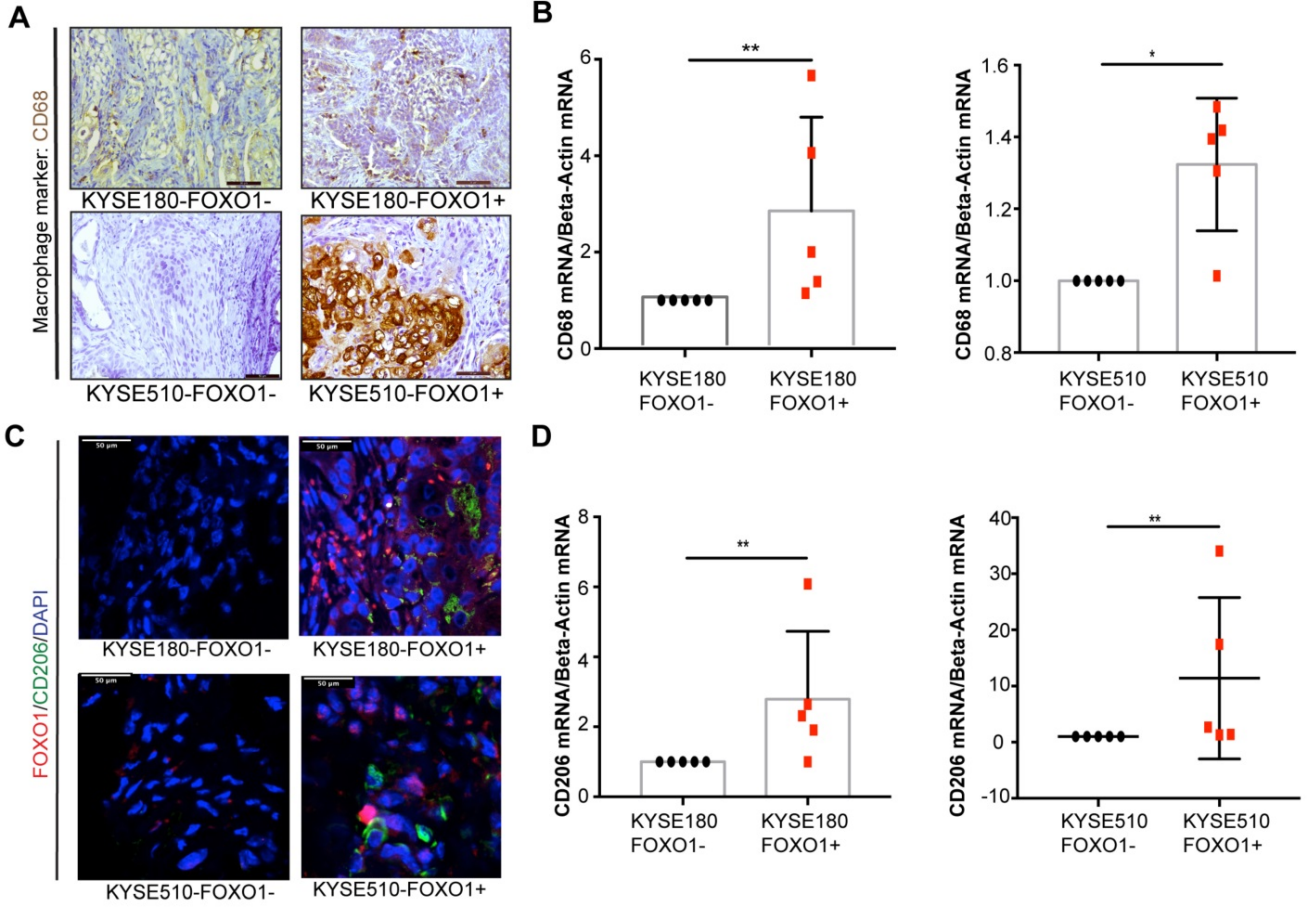
M2 macrophages infiltration in mice models. FOXO1(+) and FOXO1(-) tumor cells were injected into both flanks of nude mice and tumors were harvested after 3 weeks. (A) Representative images of IHC staining with CD68 in the FOXO1(+) group and FOXO1(-) tumor tissue. Scale bar, 50 µm. (B) Relative expression of CD68 in FOXO1(+) (n = 5) and FOXO1(-) (n = 5) groups analyzed using qRT-PCR (*P < 0.05; **P < 0.01). (C) Representative images of IF staining with FOXO1 (red) and CD206 (green) in the FOXO1(+) and FOXO1(-) group tumor tissues. Scale bar, 50 µm. (D) Relative expression of CD206 in FOXO1(+) (n = 5) and FOXO1(-) (n = 5) groups analyzed using qRT-PCR (*P < 0.05; **P < 0.01).

To further investigate the association between tumor-derived FOXO1 and M2 macrophage infiltration *in vivo*, we established FOXO1-transfected tumor cells (FOXO1(+)) and control (FOXO1(-)) cells (Figure S1E-H) and injected them subcutaneously into the left and right dorsal flanks of nude mice (n = 5), respectively. The infiltration of M2 macrophages was determined after the tumor tissues were collected and analyzed with qRT-PCR, IHC, and IF. Higher infiltration of CD68+ macrophages was observed in the FOXO1(+) group than in the FOXO1(-) group (Figure 2A). In addition, the qRT-PCR revealed overexpression of CD68 in FOXO1(+) tumor tissues, suggesting that tumor-derived FOXO1 could promote macrophage infiltration *in vivo* (Figure 2B). To identify the subtypes of infiltrating macrophages, IF and qRT-PCR were performed and the results showed that the infiltrating macrophages in FOXO1(+) tumor tissues were primarily CD206 positive (Figure 2C-D).

### FOXO1 modulates CCL20 expression

**Figure 3:**
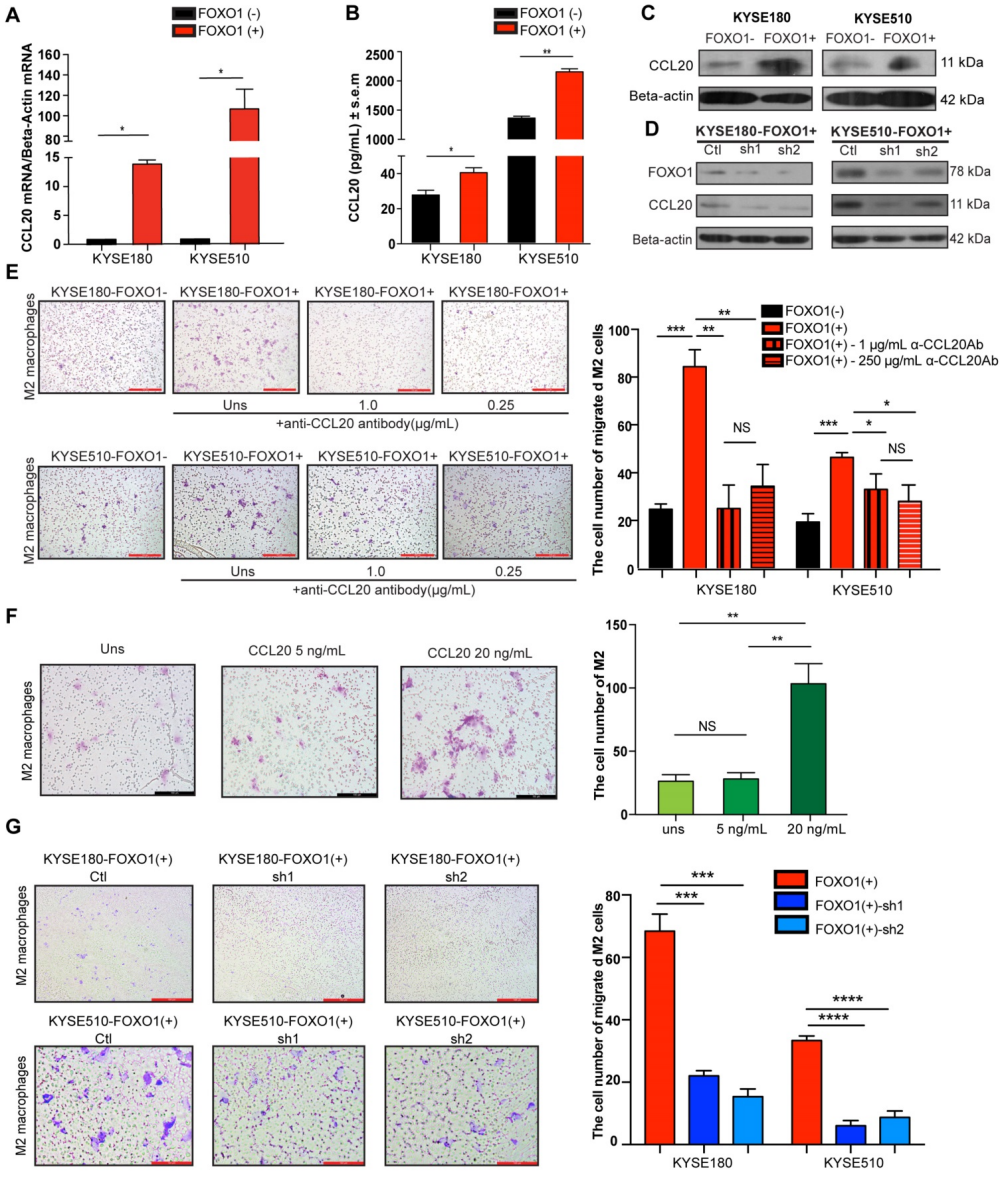
FOXO1(+) tumor cells promoted M2 macrophages recruitment by CCL20 secretion. (A) Relative expression of CCL20 in the FOXO1(+) and FOXO1(-) tumor cells detected using qRT-PCR (*P < 0.05; **P < 0.01). (B) Concentration of CCL20 in the supernatants of FOXO1(+) and FOXO1(-) tumor cells measured with ELISA (*P < 0.05; **P < 0.01). (C) Expression of protein CCL20 in FOXO1(+) and FOXO1(-) tumor cells detected with western blotting. (D) Expression of protein CCL20 in FOXO1(+) tumor cells and their FOXO1 silenced tumor cells detected with western blotting. (E) Representatives and summary of M2 macrophage migration assays induced with FOXO1(-) tumor cells, FOXO1(+) tumor cells, and FOXO1(+) tumor cells after blocking with 0.25 µg/mL and 1.0 µg/mL α-CCL20 antibody. Scale bar, 100 µm (*P < 0.05; **P < 0.01, ***P < 0.001). (F) Representatives and summary of M2 macrophage migration assays induced with serum-free medium, 5 ng/mL and 20 ng/mL CCL20 recombinant. Scale bar, 100 µm (*P < 0.05; **P < 0.01). (G) Representatives and summary of M2 macrophage migration assays induced with FOXO1(+)-Ctl tumor cells, FOXO1(+)-sh1 tumor cells, and FOXO1(+)-sh2 tumor cells. Scale bar, 100 µm (***P < 0.001; ****P < 0.0001).

A previous study reported that FOXO1 overexpression remarkably amplified NF-κB-dependent CCL20 production after TNF-α stimulation 8. Moreover, CCR6, the sole receptor of CCL20, was reported to be expressed on many myeloid lineage cells, including macrophages, dendritic cells, and monocytes. To explore whether FOXO1 affects CCL20 production, qRT-PCR, western blotting, and enzyme-linked immunosorbent assay (ELISA) were performed on FOXO1(+) and FOXO1(-) tumor cells (Figure 3A-C). The results showed that the expression of CCL20 was upregulated in FOXO1(+) tumor cells. Moreover, the upregulation of CCL20 was reversed when FOXO1 expression was silenced with FOXO1-shRNA (Figure 3D).

To confirm the correlation between FOXO1 and CCL20, we analyzed the RNA sequencing data of ESCC from TCGA database and performed Pearson's correlation between FOXO1 and CCL20 expression. The results showed that there was a positive correlation between FOXO1 and CCL20 expression in ESCC patients (r = 0.3218; 95% CI: 0.02752 to 0.5647; *P* = 0.0332) (Figure S2A). Additionally, the IHC results further validated the co-localization of FOXO1 and CCL20 in ESCC tumor tissues (Figure S2B).

### FOXO1(+) tumor cells promote the recruitment of M2 macrophages via CCL20 secretion

CCL20 has been previously reported to be capable of regulating macrophage recruitment 18. Because we showed that FOXO1 and CCL20 were positively correlated, we further hypothesized that FOXO1 could regulate M2 macrophage recruitment via a CCL20-dependent process. After polarization of THP-1 cells into M0 and M2 macrophages (Figure S2C), we performed a migration assay and the result showed that the migrated M2 macrophages were significantly increased when induced with FOXO1(+) tumor cells after 48-h incubation (Figure 3E), whereas the number of migrated M0 macrophages remained unchanged between FOXO1(+) and FOXO1(-) induction (Figure S2D). We subsequently used recombinant CCL20 protein to determine whether it could induce M2 macrophage migration. The results revealed that CCL20 recombinant significantly promoted the migration of M2 macrophages (Figure 3F). However, by introducing the CCL20 antibody, the number of migrated M2 macrophages induced by the FOXO1(+) cells was decreased (Figure 3E). Furthermore, a consistent result was observed that the migration of M2 macrophages was significantly reduced in the FOXO1-knockdown groups (FOXO1(+) sh1 and sh2 groups) (Figure 3G). These findings suggest that FOXO1 is an essential factor in mediating the migration of M2 macrophages via CCL20 secretion.

### FOXO1 facilitates M0 macrophage polarization toward M2 macrophages

**Figure 4:**
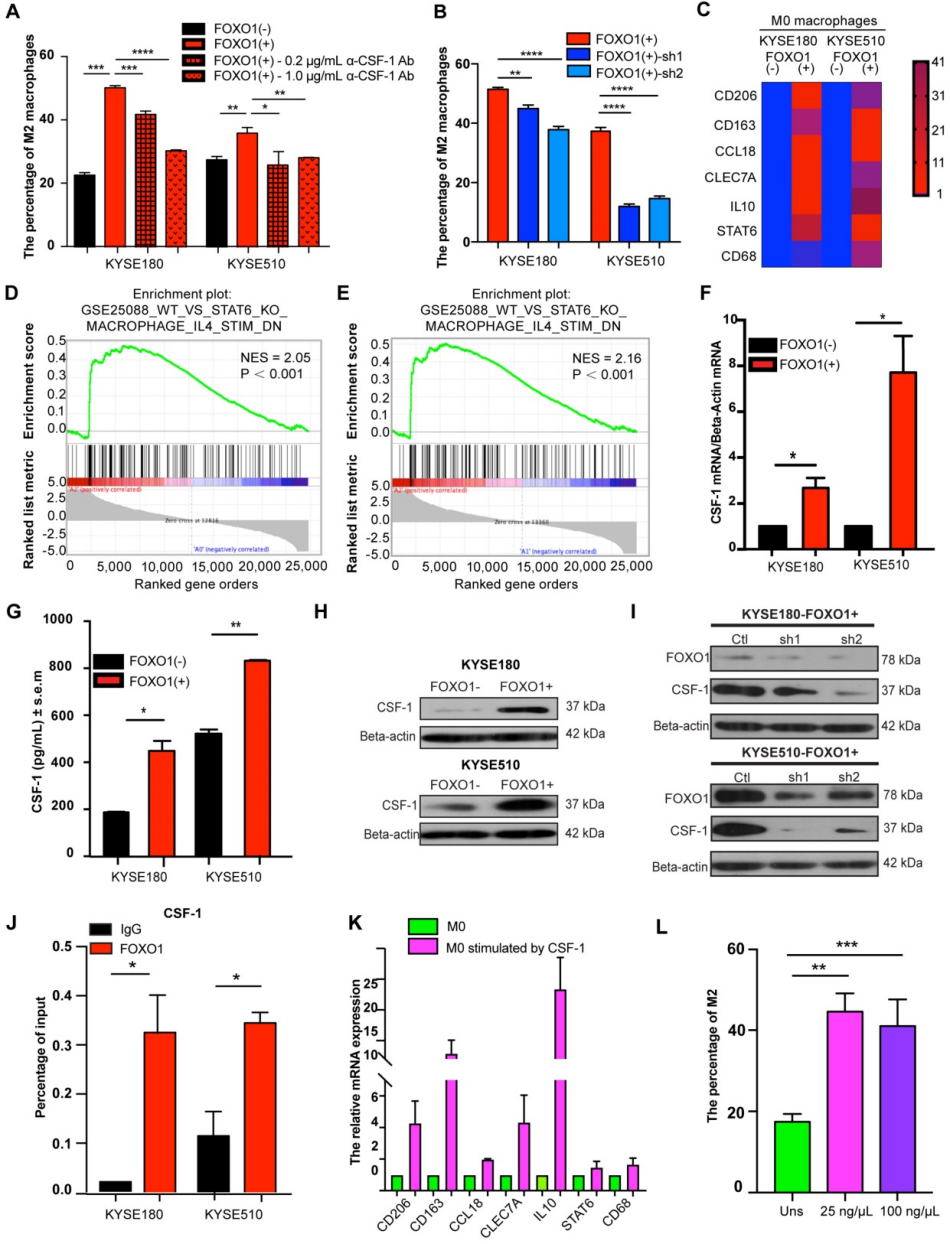
FOXO1(+) tumor cells promoted M0 macrophages differentiation towards M2 macrophages by CSF-1 production. (A) Flow cytometric analysis for CD68 and CD163 expression in M0 macrophages after co-culture with FOXO1(-) tumor cells, FOXO1(+) tumor cells, and FOXO1(+) tumor cells after blocking with 0.2 µg/mL and 1.0 µg/mL α-CSF-1 antibody. The percentage of M2 macrophages is summarized in the bar chart and the data were calculated as means ± standard error of the mean (SEM) of three independent experiments (*P < 0.05; **P < 0.01; ***P < 0.001; ****P < 0.0001). (B) Flow cytometric analysis for CD68 and CD163 expression in M0 macrophages after co-culture with FOXO1(+)-Ctl tumor cells, FOXO1(+)-sh1 tumor cells, and FOXO1(+)-sh2 tumor cells. The percentage of M2 macrophages is summarized in the bar chart and the data were calculated as means ± SEM of three independent experiments (**P < 0.01; ****P < 0.0001). (C) Heat map visualization of gene relative expression analyzed from the qRT-PCR results, which illustrated the levels of M2 macrophage markers CD206, CD163, IL10, CCL18, CLEC7A, and STAT6, and pan-macrophage marker CD68 in M0 macrophages induced with FOXO1(-) and FOXO1(+) tumor cells. Blue represents downregulation and red indicates upregulation. Color intensity reflects the mRNA expression values. (D-E) GSEA analysis was conducted and revealed the gene set of GSEA_WT_VS_STAT6_KO_MACROPHAGE_IL4_STIM (Detailed information in http://software.broadinstitute.org/gsea/msigdb/cards/GSE25088_WT_VS_STAT6_KO_MACROPHAGE_IL4_STIM_DN) was enriched in FOXO1(+) tumor cell-induced M0 group when this group was compared with the initial M0 group (Normalized Enrichment Score (NES) = 2.05, *P* < 0.001) (D) or FOXO1(-) tumor cell-induced M0 group (NES = 2.16, *P* < 0.001) (E). (F) Relative expression of CSF-1 in FOXO1(+) and FOXO1(-) tumor cells detected using qRT-PCR (*P < 0.05; **P < 0.01). (G) Concentration of CSF-1 in the supernatants of FOXO1(+) and FOXO1(-) tumor cells measured using ELISA (*P < 0.05; **P < 0.01). (H) Expression of protein CSF-1 in FOXO1(+) and FOXO1(-) tumor cells detected with western blot analysis. (I) Expression of protein CSF-1 in FOXO1(+) tumor cells and their FOXO1 silenced tumor cells detected using western blot analysis. (J) ChIP-qPCR analysis of FOXO1 binding to CSF-1 promoter. Precipitated DNAs were quantified with qPCR for promoter regions of CSF-1 gene (*P < 0.05). (K) Relative expression of M2 macrophage markers in M0 macrophages and M0 macrophages stimulated with CSF-1 recombinant (100 µg/µL) detected using qRT-PCR (*P < 0.05; **P < 0.01). (L) Flow cytometric analysis for CD68 and CD163 expression in M0 macrophages stimulated with PBS, 25 µg/µL CSF-1, and 100 µg/µL CSF-1 recombinant. The percentage of M2 macrophages is summarized in the bar chart and the data were calculated as means ± SEM of three independent experiments (**P < 0.01; ***P < 0.001).

We established a co-culture system with ESCC tumor cells and M0 macrophages. The flow cytometry results showed that the percentage of M2 macrophages (CD163+/CD68+) was increased when M0 macrophages were co-cultured with FOXO1(+) tumor cells (Figure 4A and Figure S3A). Moreover, the ability of FOXO1-knockdown tumor cells to induce M2 polarization was impaired (Figure 4B and Figure S3B). To more comprehensively define the phenotypes of the induced M2 macrophages, further M2 macrophage markers were analyzed using qRT-PCR. We found that FOXO1-induced M2 macrophages expressed significantly higher levels of CD206, CD163, IL10, CCL18, CLEC7A, and STAT6 (Figure 4C). The results illustrated that FOXO1 could induce the polarization from M0 to M2 macrophages. RNA sequencing was also performed in FOXO1(+) KYSE180 cells-induced M0 macrophages, FOXO1(-) KYSE180 cell-induced M0 macrophages, and non-induced M0 macrophages. The genetic profile and clustering based on differentially expressed genes (DEGs) revealed that FOXO1(+)-induced M0 macrophages possessed a genetic profile that was highly distinct from both FOXO1(-)-induced M0 and non-induced M0 macrophages (Figure S3C-D). Additionally, Gene Set Enrichment Analysis (GSEA) exhibited that the FOXO1(+)-induced M0 macrophages displayed similar molecular signatures with IL4-stimulated M2 macrophages reported by Attila Szanto et al. 19 (Figure 4D, E). We later confirmed the overexpression of CD206, CD163, IL10, CCL18, CLEC7A, and STAT6 in IL4 and IL13-stimulated M2 macrophages (Figure S3E), indicating that the FOXO1-induced macrophages were M2 macrophages.

### CSF-1 mediates FOXO1-transfected cell-induced M0 macrophages

CCL20 was the first cytokine that we suspected as a downstream factor that was involved in the FOXO1-mediated M0-to-M2 polarization. However, we found that the recombinant CCL20 protein did not significantly influence the expression of M2 markers CCL18, CD206, and CD163 in M0 macrophages (Figure S4A). Because IL4, IL13, IL10, and CSF-1 are stimuli in M2 differentiation as reported in previous studies 6, 20, we performed qRT-PCR in FOXO1(+) and FOXO1(-) tumor cells to detect the expression of these genes (Figure S4B). We found that CSF-1 expression was significantly upregulated in FOXO1(+) tumor cells (Figure 4F). The overexpression of CSF-1 was also validated using ELISA and western blotting (Figure 4G-H).

CSF-1, which is known as one of the colony-stimulating factors, can regulate the development of trophoblast lineage cells and the mononuclear phagocyte system via its receptor CSF-1R. CSF-1 has been reported to induce the polarization of monocytes towards M2 macrophages with high CD206 and CD163 expression 21-23. To further determine the relationship between FOXO1 and CSF-1, FOXO1 shRNA and its negative control plasmid were transfected into FOXO1(+) tumor cells. The overexpression of CSF-1 was found to decrease after FOXO1 silencing (Figure 4I). The ChIP-qPCR assay was also conducted to identify the transcriptional alteration and the result revealed that the FOXO1 antibody efficiently precipitated CSF-1 promoter fragments compared to the IgG control (Figure 4J). Subsequently, we co-cultured FOXO1(+) tumor cells with M0 macrophages in the presence of the anti-CSF-1 antibody and found that a percentage of the CD163+/CD68+ macrophages was significantly reduced (Figure 4A and Figure S3A). Furthermore, the qPCR result showed that CD206, CD163, and CCL18 expression were upregulated after CSF-1 stimulation (Figure 4K) and the flow cytometry results also validated that CSF-1 increased the percentage of CD163+/CD68+ cells. (Figure 4L). Altogether, these results suggested that FOXO1 induced M0 macrophages to differentiate into M2 macrophages via a CSF-1-dependent mechanism.

### FOXO1 promotes tumor progression by M2 macrophages via the FAK/PI3K/AKT pathway

M2 macrophages can secrete tumor-promoting factors, including Arg-1, TGF-b, and CCL18, which are proficient in tumor initiation and progression. To explore the tumor-promoting effects of M2 macrophages on tumor cells, we collected and used the M2 conditioned medium to stimulate ESCC tumor cells.

**Figure 5:**
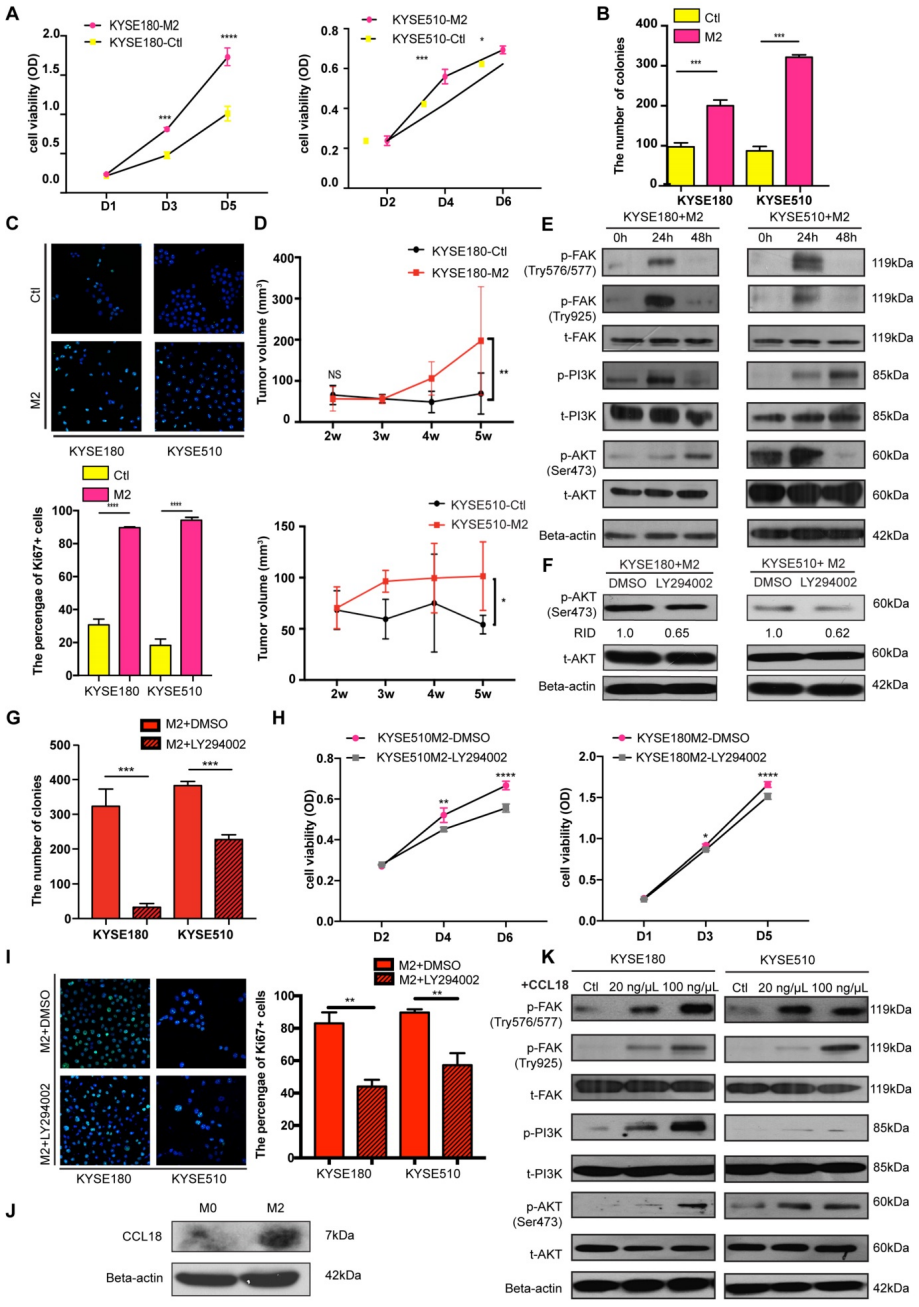
M2 macrophages promoted tumor cell proliferation and migration via the FAK/PI3K/AKT transduction signal pathway. (A) Cell viability of tumor cells detected using the XTT assay after treatment of M2 conditioned medium or control medium (*P < 0.05; ***P < 0.001; ****P < 0.0001). (B) Foci formation assay of tumor cells was conducted using M2 conditioned medium and control medium (***P < 0.001). The numbers of foci were calculated and are shown in the bar chart. (C) Representative images of IF showed the number of Ki67+ tumor cells after treatment of M2 conditioned medium and control medium (****P < 0.0001). The numbers of Ki67+ tumor cells were calculated and are shown in the bar chart. (D) The tumor volumes of excised tumors from mice injected with tumor cells stimulated by M2 conditioned medium and control medium. Linear graphs illustrate the growth rate of tumor after injection (n = 5 mice per group) (*P < 0.05; **P < 0.01). (E) Western blot results show the transduction signal pathway. The FAK-PI3K-AKT pathway was activated when tumor cells were stimulated by M2 conditioned medium. (F) Western blot results show the phosphorylation of AKT when tumor cells were treated with DMSO or LY294002 after being stimulated using M2 conditioned medium (RID: Relative Integrated Density). (H) Cell viability of tumor cells detected with the XTT assay after treatment of M2 conditioned medium combined with DMSO or LY294002 (*P < 0.05; **P < 0.01; ****P < 0.0001). (G) Foci formation assay of tumor cells was conducted using M2 conditioned medium with/without DMSO or LY294002 (***P < 0.001). The numbers of foci were calculated and are shown in the bar chart. (I) Representative images of IF showed the number of Ki67+ tumor cells after the treatment of the M2 conditioned medium with/without DMSO or LY294002 (**P < 0.01). The numbers of Ki67+ tumor cells were calculated and are shown in the bar chart. (J) Western blot results show the expression of CCL18 and Beta-actin in M0 macrophages and M2 macrophages. (K) Western blot results show the activation of the FAK-PI3K-AKT pathway when tumor cells were stimulated by PBS and 20 ng/µL and 100 ng/µL CCL18 recombinant.

The functional assays revealed that the tumor cells had a higher proliferation rate when cultured with the M2 conditioned medium (Figure 5A). M2 conditioned medium also enhanced the ability of tumor cells to form colonies, as indicated from the foci formation assay (Figure 5B and Figure S5A). Ki67, an important marker for cell proliferation, was also detected with IF and the result revealed that more Ki67(+) tumor cells were present in the M2 macrophages-stimulated group than in the control group (Figure 5C). Additionally, in the co-culture experiment between FOXO1(+)/(-) tumor cells and M0 macrophages, the FOXO1(+) group showed a higher percentage of Ki67(+) tumor cells than the FOXO1(-) group (Figure S5B). Therefore, M2 macrophages significantly promoted tumor cell proliferation. Besides proliferation, M2 conditioned medium also facilitated tumor cell migration. The result of the migration assay showed that M2 macrophages enhanced the migratory and invasive potential of tumor cells (Figure S5C-E). To further validate the tumor-promoting effects of M2 macrophages *in vivo*, we injected parental ESCC tumor cells into the flanks of mice subcutaneously. After two weeks, we injected concentrated M2 macrophage conditioned medium and serum-free medium into the mice subcutaneously every three days. After five weeks, we observed that the tumor size in the M2 conditioned medium-treated group was significantly larger than the size in the control group (Figure 5D and Figure S5F).

The FAK/PI3K/AKT transduction signal pathway has been previously reported to be involved in tumor migration and proliferation induced by TAMs 24-27. Therefore, we investigated whether the M2 conditioned medium could activate this pathway during TAM-tumor interaction. Upon treatment with the M2 conditioned medium, enhanced FAK, PI3K, and AKT phosphorylation was detected using western blotting (Figure 5E). To further confirm the M2-mediated activation, we applied PI3K inhibitor LY294002 to block the pathway and found that LY294002 inhibited the activation of AKT (Figure 5F). Moreover, after treatment with LY294002, the potential of colony formation induced by the M2 conditioned medium was reduced (Figure 5G and Figure S5G). The XTT proliferation assay showed a consistent result, whereby LY294002 impeded the M2-induced proliferation in tumor cells (Figure 5H). In the presence of LY294002, a decreasing percentage of Ki67(+) tumor cells in the M2 macrophages-stimulated group was observed (Figure 5I). This result validated that the FAK/PI3K/AKT pathway was activated by M2 macrophages to promote tumor proliferation.

Accumulating evidence shows that CCL18 is involved in the FAK/PI3K/AKT pathway and epithelial-mesenchymal transition (EMT) in various types of cancer 28, 29. Hence, we collected the medium from M0, M2, and FOXO1(+)/(-)-induced M0 macrophages to detect the concentration of CCL18. Results from the western blot analysis showed that CCL18 secretion was upregulated in M2 macrophages (Figure 5J and Figure S5H). Then, we cultured parental tumor cells with different concentrations of CCL18 recombinant protein and found that the FAK-PI3K-AKT pathway was activated (Figure 5K). Although the interplay between M2 macrophages and tumor cells is a complicated mechanism involving various factors, these results suggested that CCL18 secreted by M2 macrophages was one of the factors associated with the interaction.

## Discussion

TAMs have been well-recognized as tumor-promoting macrophages that contribute to tumor development and metastasis. Although TAMs are an ontogenetically heterogeneous population, they exhibit many similar characteristics to M2 macrophages, including an anti-inflammatory phenotype and the state of activation and localization 20. TAMs exert immunosuppressive effects on tumor-infiltrating lymphocytes and secrete anti-inflammatory cytokines and chemokines, such as CCL18 or Arg1, which directly enhance tumor progression 7, 30. In breast cancer, CCL18 produced by TAMs has been reported to promote cancer metastasis via PITPNM3-dependent calcium signaling activation 24. In addition, cytokines secreted by TAMs, including TGF-β and IL10, have been found to affect PD-1/PD-L1 expression 31. Patients with a high density of TAMs in the TME are correlated with worse survival 32. Although the function of TAMs is well elucidated and its prognostic value has been validated in various cancers, the infiltration of macrophages is not a prevalent feature in cancer patients. Only 33% of urinary bladder cancers have high macrophage infiltration 33 and, in breast cancer, prominent macrophage infiltration is present in 56.4% of tumor nests and 65% of the tumor-stromal area 34. Owing to the different infiltration degrees of macrophages, only patients with a high density of macrophages have a chance to benefit from TAM-targeted therapy. Therefore, the identification and characterization of tumor-derived factors that induce the infiltration of TAMs are urgently required to unveil the mechanism underlying the tumor-macrophage interaction.

In the present study, we found that FOXO1 induced the infiltration of M2 macrophages and led to poor prognosis in ESCC patients. In our clinical cohort, we showed that FOXO1 was highly upregulated in tumor tissues compared to the adjacent non-tumor counterparts. The FOXO1 overexpression often occurred at the tumor margin adjacent to stromal tissues. Transcription factor FOXO1 was reported to modulate the production and secretion of various cytokines and chemokines; thus, we hypothesized that FOXO1 in ESCC might also interact with macrophages via a transcriptional network. In the present study, we found that M2 macrophages were frequently located in the tumor-stroma region, where most of the tumor cells overexpressed FOXO1. This phenomenon was further confirmed by the *in vivo* experiments in which the number of infiltrating M2 macrophages was significantly increased when co-cultured with FOXO1(+) tumor cells. By excluding the possibility that FOXO1 directly mediated tumor progression (Figure S5A-B), we hypothesized that FOXO1 might induce the activation of M2 macrophages to indirectly enhance tumor progression. The subsequent experiments validated that FOXO1(+) tumor cells promoted M0-to-M2 polarization and the migration of M2 macrophages. A dramatically higher expression of CCL20 was detected in FOXO1(+) tumor cells, suggesting CCL20 was the downstream factor that was responsible for the recruitment of M2 macrophages. Additionally, another cytokine CSF-1 modulated by FOXO1 was found to be crucial to M0-to-M2 polarization 20. Our data suggested both CCL20-mediated recruitment of M2 macrophages and CSF-1-induced polarization synergistically contributed to the enhanced infiltration of M2 macrophages in FOXO1-overexpressing tumors. Upon treatment with the conditioned medium from M2 macrophages, tumor cells displayed rapid growth both *in vivo* and *in vitro*. Subsequent investigation was conducted to decipher the molecular mechanism underlying the interactive effects of M2 macrophages on tumor cells and vice versa. As previously reported in colorectal cancer 35 and lung cancer 36, the FAK-PI3K-AKT signal transduction pathway is closely correlated with tumor proliferation and patient survival. Using the M2 conditioned medium, we found that the FAK-PI3K-AKT signal transduction pathway was activated in tumor cells. Among the various factors in the M2 condition medium, we identified CCL18 as one of the tumor-promoting cytokines involved in FAK-PI3K-AKT activation.

Accumulating evidence has proven the tumor-promoting effects of M2 macrophages in the TME; therefore, targeting M2 macrophages might provide a promising therapeutic strategy in cancer treatment. In the present study, FOXO1 was reported to facilitate the infiltration of M2 macrophages by modulating chemoattractant CCL20 and CSF-1 expression. The overexpression of FOXO1 in tumor cells indicated a high density of M2 macrophages in the tumor-stromal region and a worse prognosis in ESCC patients. The identification of FOXO1 could stratify M2 macrophage-infiltrated ESCC patients and offer more personalized treatment for patients showing high M2 macrophage infiltration. Pharmacological inhibition of CCL20 and CSF-1 or the genetic silencing of FOXO1 might serve as an excellent candidate to suppress tumor progression and improve prognosis. In summary, FOXO1 is a potential prognostic factor that has a tumor-promoting effect on the infiltration of M2 macrophages and the polarization of M0 macrophages. Together, these data shed light on the understanding of the mechanism of tumor-macrophage interaction and are likely to stimulate further advances in prognostic and therapeutic developments in ESCC.

## Supplementary Material

Supplementary figures and tables.

## Figures and Tables

**Table 1:** The clinicopathological characteristics based on FOXO1 expression

	Total (n = 144)	Foxo1 expression		*P*
		Negative (n = 96)	Positive (n = 48)	
**Gender**				0.906
Male	74	49	25	
Female	70	47	23	
**Age**	60.9 (40~80)	60.5 (40~80)	61.6 (40~79)	0.724
<60		49	23	
≥60		47	25	
**Pathological type**				0.158
medullary	72	42	30	
fungating	13	11	2	
ulcerative	38	28	10	
others	21	15	6	
**Invasion**				0.765
1	12	8	4	
2	9	5	4	
3	123	83	40	
**Differentiation**				0.313
well	14	11	3	
modest	98	61	37	
poor	30	22	8	
**pT stage**				0.606
pT1	13	9	4	
pT2	40	29	11	
pT3	91	58	33	
**pN stage**				0.185
pN0	64	46	18	
pN1	77	47	30	
pN2	3	3	0	
**Distant metastasis**				0.013
M0	141	96	45	
M1	3	0	3	

pT stage: pathological T stage; pN stage: pathological N stage.

**Table 2:** Univariate and multivariate Cox regression analysis

Variable	univariate			multivariate		
	Hazard ratio	95%CI	*P*	Hazard ratio	95%CI	*P*
**Gender**						
Male	1			1		
Female	0.876	0.550-1.394	0.575	0.966	0.540-1.727	0.907
**Age**						
<60	1			1		
≥60	1.801	1.121-2.894	0.015	0.522	0.286-0.954	0.035
**Pathological type**			0.429			0.539
medullary	1			1		
fungating	1.519	0.732-3.154	0.262	1.034	0.350-3.055	0.952
ulcerative	1.184	0.397-3.532	0.762	2.309	0.545-9.794	0.256
others	1.858	0.855-4.038	0.118	0.942	0.304-2.923	0.918
**Invasion**			0.09			0.906
1	1			1		
2	0.207	0.051-0.846	0.028	<0.001	0-4.42e+18	0.718
3	<0.001	1.57E+251	0.965	0.001	0-1.05e+21	0.796
**Differentiation**			0.015			0.422
well	1			1		
modest	0.317	0.124-0.812	0.017	0.349	0.072-1.697	0.192
poor	1.405	0.836-2.359	0.199	0.85	0.453-1.595	0.613
**pT stage**			<0.001			0.074
pT1	1			1		
pT2	0.175	0.043-0.718	0.016	6.33	0.723-55.39	0.095
pT3	0.274	0.136-0.553	<0.001	0.569	0.245-1.322	0.19
**pN stage**			<0.001			<0.001
pN0	1			1		
pN1	275.48	0-3.738e+48	0.917	152.93	0-2185e+63	0.944
pN2	12179.35	0-1.657e+50	0.862	9587.64	0-1.361e+65	0.898
**Distant metastasis**						0.561
M0	1			1		
M1	2.517	0.788-8.043	0.119	1.473	0.399-5.434	0.561
**FOXO1**						
negative	1			1		
positive	0.577	0.340-0.980	0.042	0.539	0.300-0.970	0.039

## Abbreviations

CAF: cancer associated fibroblast
CCL20: chemokine ligand 20
cDNA: complementary DNA
CSF-1: colony stimulating factor 1
CM: complete medium
ECL: enhanced chemiluminescence
ELISA: enzyme-linked immunosorbent assay
ESCC: esophageal squamous cell carcinoma
EMT: epithelial mesenchymal transition
FBS: fetal bovine serum
FOXO: forkhead box O
HRP: horseradish peroxidase
IF: immunofluorescence
IHC: immunohistochemistry
OD: optical density
OS: overall survival
PFA: paraformaldehyde
PMA: phorbol 12-myristate 13-acetate
qRT-PCR: quantitative real-time polymerase chain reaction
TAM: tumor associated macrophage
TMA: tissue microarray
TCGA: The Cancer Genome Atlas
