# Supplementary material for: FOXO1 promotes tumor progression by increased M2 macrophage infiltration in esophageal squamous cell carcinoma
Source: Theranostics. 2020 Sep 16;10(25):11535–48. doi: 10.7150/thno.45261 (PMC7546008; doi:10.7150/thno.45261)
Supplement: Supplementary file 1 — Supplementary figures and tables. [file thnov10p11535s1.pdf]

Supplemental Figures

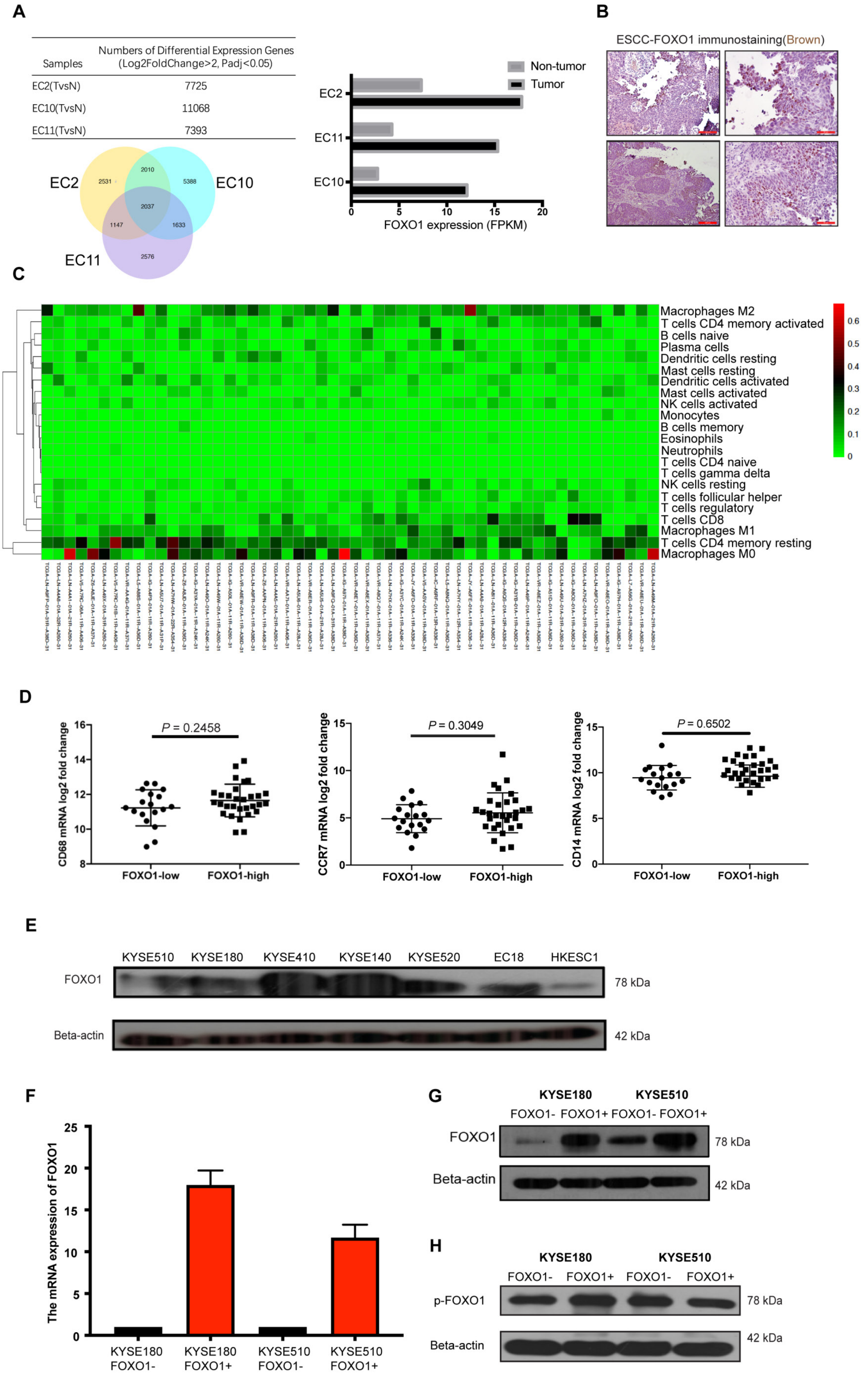

Figure 1. The expression of FOXO1 and the infiltration of immune cells. (A) FOXO1 was upregulated in tumor tissues in RNA sequencing data of three paired ESCC tumor and non-tumor tissues (B) Representative images of IHC staining by FOXO1 (brown) in the FOXO1-positive tumor tissue. The FOXO1 overexpression often occurred at tumor edge adjoining stromal tissues. Scale bar, left 200 μm and right 50 μm. (C) The heat map of 22 types of immune cells infiltrating the ESCC tumor tissues analyzed in TCGA database. The columns represent each patient sample and the proportions of the immune cells are shown as the color intensity. Red represents high density and green indicates low density. (D) The mRNA expression of CD68 was no significant different between FOXO1-high (n = 30) and FOXO1-low groups (n = 17) although it displayed an upregulated trend in the FOXO1-high group ($P = 0.2485$). The mRNA expression of CCR7 was no significant different between FOXO1-high and FOXO1-low groups ($P = 0.3049$). The mRNA expression of CD14 was no significant different between FOXO1-high and FOXO1-low groups ($P = 0.6502$). (E) Western blot results showed the expression of FOXO1 and Beta-actin in ESCC cell lines. (F) The relative expression of FOXO1 in FOXO1(-) and FOXO1(+) tumor cells detected by qRT-PCR. (G) Western blot results showed the expression of FOXO1 and Beta-actin in in FOXO1(-) and FOXO1(+) tumor cells. (H) Western blot results showed the phosphorylation of FOXO1 and Beta-actin in FOXO1(-) and FOXO1(+) tumor cells.

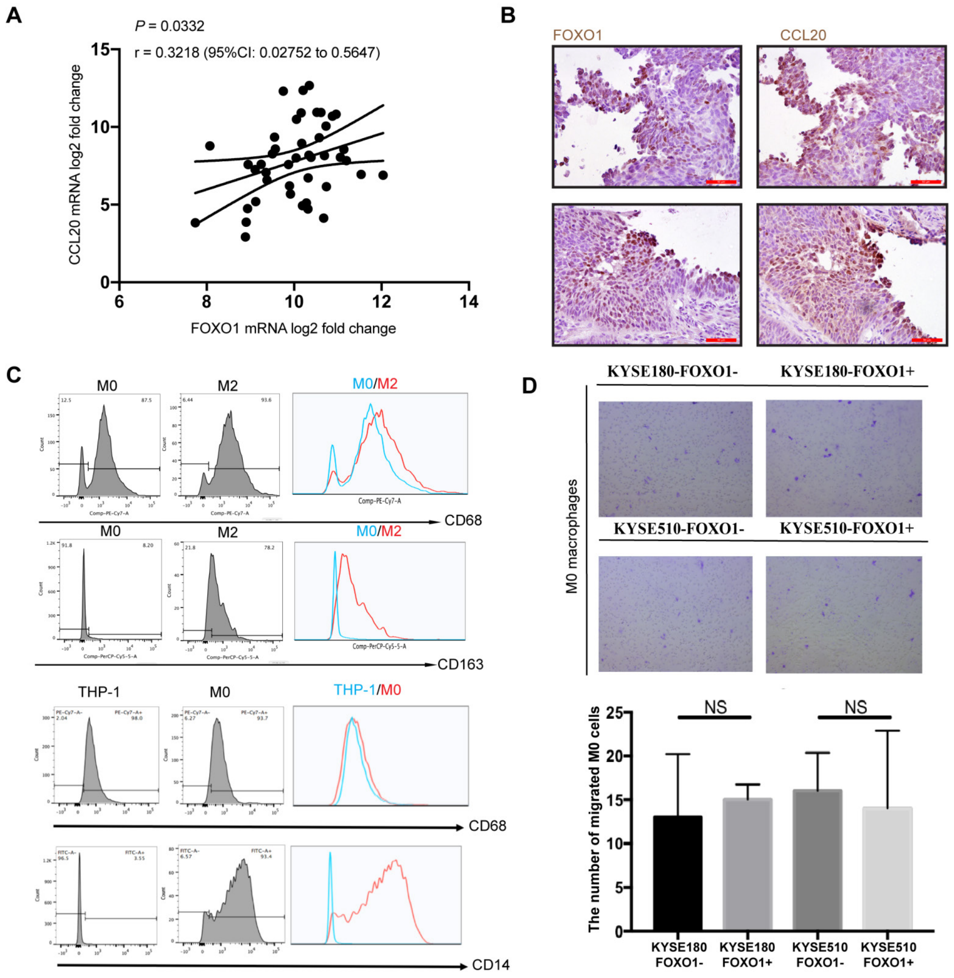

Figure 2. The expression of CCL20 and the phenotypes of induced M2 macrophages and M0 migration assay induced by FOXO1(+) and FOXO1(-) tumor cells. (A) Pearson's correlation coefficient (R) analysis of CCL20 and FOXO1 of ESCC patients in TCGA database. A modest positive correlation was between FOXO1 and CCL20 expression (n = 46) (r = 0.3218; 95%CI: 0.02752 to 0.5647; *P* = 0.0332) (B) Representative images of IHC staining by FOXO1(left) and CCL20 (right) in the same FOXO1-positive tumor tissues. The cells overexpressing CCL20 were always located in the area where the cells overexpressing FOXO1 Scale bar, 50 μm. (C) The expression of CD68, CD14 and CD163 detected by flow cytometry. (D) The migrated M0 macrophages induced by FOXO1(-)/FOXO1(+) tumor cells (NS, no significant difference).

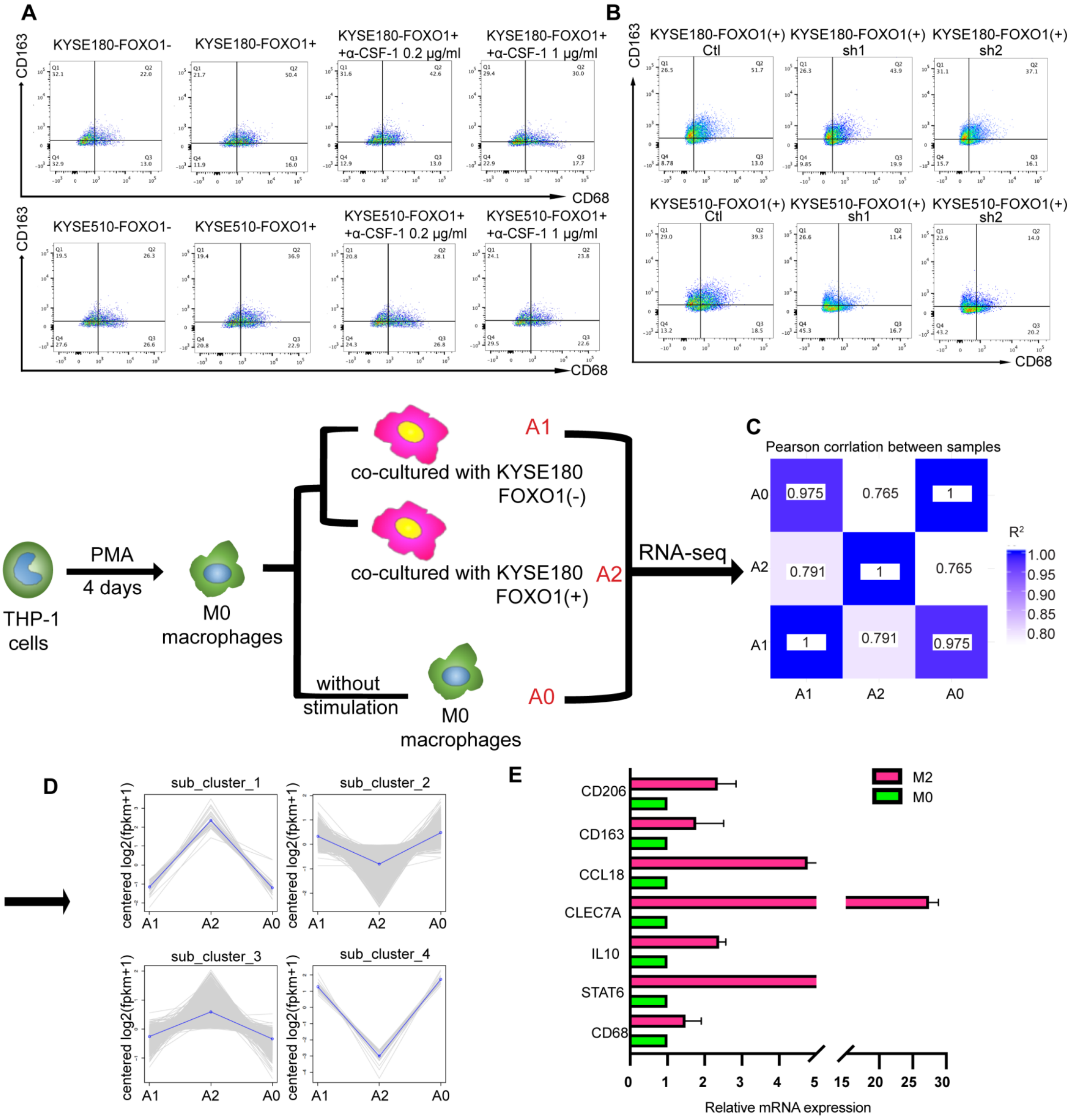

Figure 3. The phenotypes of FOXO1-induced macrophages. (A) Flow cytometric images of CD68 and CD163 expression in M0 macrophages after co-culture with FOXO1(-) tumor cells, FOXO1(+) tumor cells, and FOXO1(+) tumor cells after blocking with 0.2 μg/mL and 1.0 μg/mL α-CSF-1 antibody. (B) Flow cytometric images of CD68 and CD163 expression in M0 macrophages after co-culture with FOXO1(+)-Ctl tumor cells, FOXO1(+)-sh1 tumor cells, and FOXO1(+)-sh2 tumor cells. (C) The analysis of RNA sequencing conducted among initial M0 macrophages and M0 macrophages induced by FOXO1(-) and FOXO1(+) tumor cells. The heap map illustrates the correlation between two groups. Blue represents high correlation while white indicates low correlation. Color intensity reflects degree of relevancy. The analysis was conducted by Pearson's chi-square test. (D) Clustering analysis of DEGs between three groups (initial M0 macrophages, M0 macrophages induced by

FOXO1(-) and FOXO1(+) tumor cells) revealed expression patterns of genes in four main clusters. Gray lines indicate relative expression of a gene cluster and the blue line represents the level of consensus of all the genes in the cluster. (E) The relative expression of M2 major markers (CD206, CD163, CLEC7A, STAT6, IL10 and CCL18) and CD68 in M0 macrophages and IL4 and IL13-induced M2 detected by qRT-PCR.

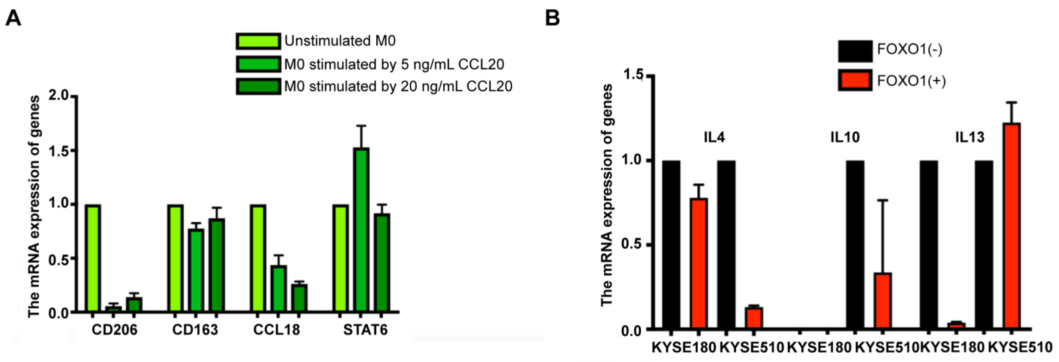

Figure4. The expression of genes. (A)The relative expression of M2 major markers (CD206, CD163, STAT6 and CCL18) in M0 macrophages and M0 macrophages stimulated by CCL20 recombinant (5 ng/mL and 20 ng/mL) (B) The relative expression of FOXO1 downstream molecules (IL4, IL13 and IL10) in FOXO1(-) and FOXO1(+) tumor cells.

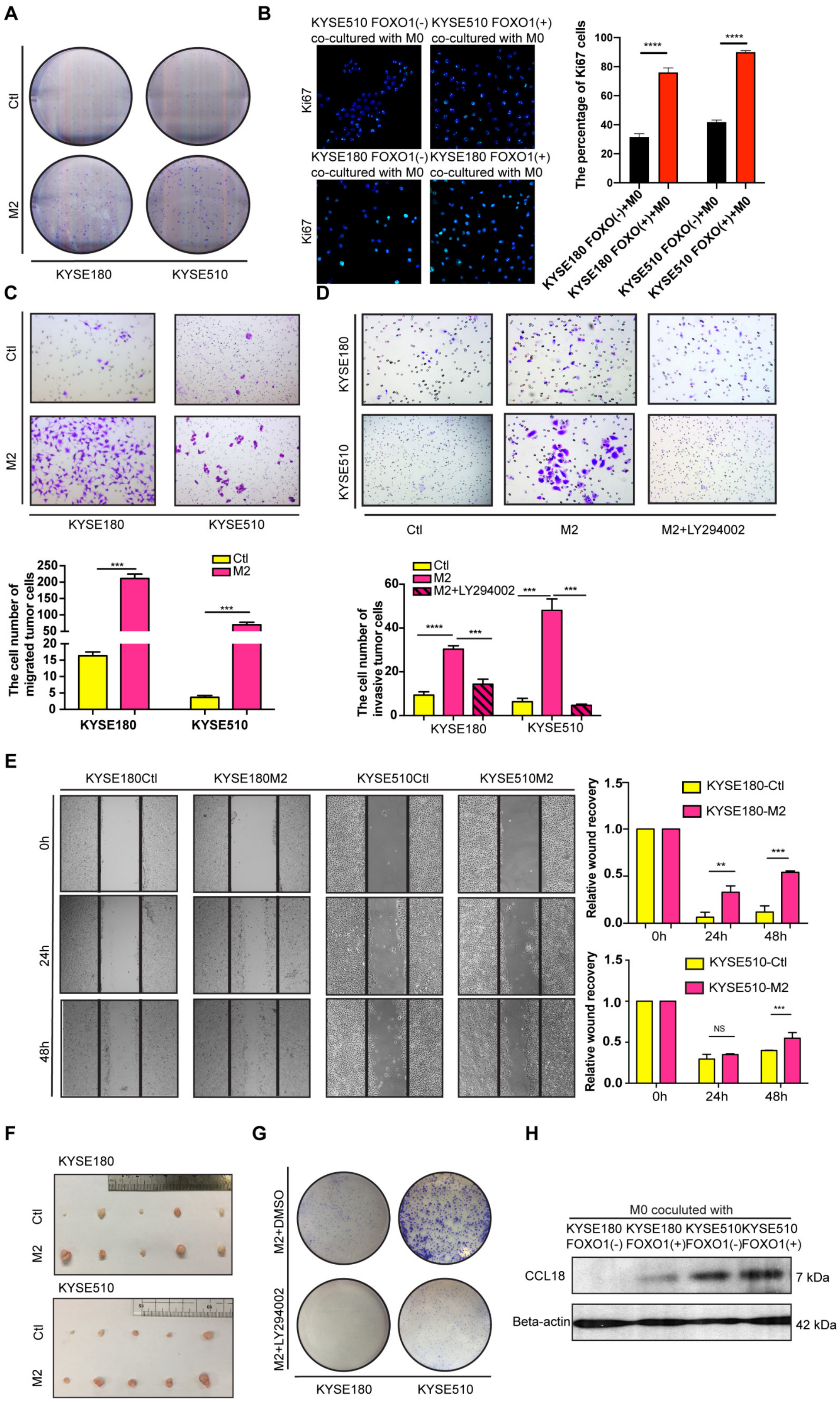

Figure5 (A) Representative images of foci formation assay of tumor cells treated with M2 conditioned medium and control medium. (B) Representative images of IF showed the number of Ki67+ tumor cells in FOXO1(+) and FOXO1(-) groups after they

co-cultured with M0 macrophages. (*****P* < 0.0001). The numbers of Ki67+ tumor cells were calculated and shown in the bar chart. (C) The migration assay of tumor cells induced by M2 conditioned medium and control medium (****P* < 0.001). The numbers of migrated tumor cells were calculated and shown in the bar chart. (D) The invasion assay of tumor cells induced by control medium, M2 conditioned medium with DMSO and LY294002 (****P* < 0.001; *****P* < 0.0001). The numbers of migrated tumor cells were calculated and shown in the bar chart. (E) The wound healing experiment displayed tumor cell motility induced by M2 conditioned medium and the control medium (NS, no significant difference; ***P* < 0 .01; ****P* < 0.001). Black Lines indicate the initial wound borders. The scratch area was measured by ImageJ and relative wound recovery was calculated as the ratio of the remaining scratch area and the initial scratch area at 0 hour. (F) Representative images of excised tumors from mice injected with tumor cells stimulated by M2 conditioned medium and control medium. (G) Representative images of foci formation assay of tumor cells induced by M2 conditioned medium with/without DMSO or LY294002 (****P* < 0.001). (H) Western blot results showed the expression of CCL18 and Beta-actin in M0 macrophages induced by FOXO1(-) and FOXO1(+) tumor cells.

Supplementary Table 1

Primers	Forward primer (5' to 3')	Reverse primer (5' to 3')
FOXO1	GGGTTAGTGAGCAGGTTACAC	TCCAATGGCACAGTCCTTATC
IL10	GCTGGAGGACTTTAAGGGTTAC	TGCCTTTCTCTTGGAGCTTATT
CCL20	GCAAGCAACTTTGACTGCTG	CAAGTCCAGTGAAGGCACAAA
MMP9	CAGTACCGAGAGAAAGCCTATTT	CCTTTCCTCCAGAACAGAATACC
CCL18	GGTGTCATCCTCCTAACCAAGA	GGCATAGCAGATGGGACTCT
CD206	ACGATCCGACCCTTCCTTGA	GCTTGCAGTATGTCTCCGCT
CD163	GCGGCTTGCAGTTTCCTCAA	TCCTTTTCAGTGTGGCTCAGA
Fibronectin	GACCTATCCAAGCTCAAGTGGT	TCCAAGGTTTCTGGGTGGGA
CSF-1	TGCTGTTGTTGGTCTGTCTC	GGTAGCACACTGGATCTTTCAA
CD68	CCCACCTGCTTCTCTCATTC	CGAGAATGTCCACTGTGCT
MMP2	GATACCCCTTTGACGGTAAGGA	CCTTCTCCCAAGGTCCATAGC
MMP12	TACACATTCAGGAGGCACAAA	CACGGTAGTGACAGCATCAA
STAT6	GTTCCGCCACTTGCCAATG	TGGATCTCCCCTACTCGGTG
CLEC7A	GGAAGCAACACATTGGAGAATGG	AGAACCCCTGTGGTTTTGACA
ARG1	GTGGAAACTTGCATGGACAAC	AATCCTGGCACATCGGGAATC
Beta-actin	CATCCACGAAACTACCTTCAACTCC	GAGCCGCCGATCCACACG

Supplemental Table 2. The list of antibodies

Antibody	Company
Beta-actin	Abcam
CCL20	Abcam
Snail	Cell Signaling Technology
CSF-1	Cell Signaling Technology
E-cadherin	Cell Signaling Technology
Vimentin	Cell Signaling Technology
phospho-FAK (Tyr576/577)	Cell Signaling Technology
phospho-FAK (Try925)	Cell Signaling Technology
FAK	Cell Signaling Technology
p-PI3K	Cell Signaling Technology
phospho-AKT	Cell Signaling Technology
AKT	Cell Signaling Technology
CD68-PECy7	BD Biosciences
CD14-FITC	BD Biosciences
CD163-PerCP5.5	BD Biosciences
CD68 (Mouse anti-human)	Invitrogen
CD68 (rabbit anti-mouse/human)	Abcam
CD206	Abcam
Ki-67	Abcam
Beta-catenin	Cell Signaling Technology
p-FOXO1	Abcam
